# Male involvement in maternal and child nutrition in low-income informal settlements, Nairobi, Kenya

**DOI:** 10.1186/s41043-023-00476-1

**Published:** 2024-04-04

**Authors:** Ann W. Muthiru, Salome A. Bukachi

**Affiliations:** Department of Anthropology, Gender, and African Studies (DAGAS), University of Nairobi (UON), Nairobi, Kenya

**Keywords:** Beliefs and values, Male involvement, Maternal and child nutrition, Socio-ecological model

## Abstract

**Background:**

Maternal and child nutrition is a significant public health concern because adequate nutrition is essential for the health, development, and well-being of mothers and children. Men can play a critical role in improving maternal and child health, including through their involvement in the nutrition of the mother and child. However, little has been studied on male involvement in maternal and child nutrition; therefore, this qualitative exploratory study focused on the level of male involvement and factors influencing male involvement in maternal and child nutrition in low-income urban informal settings.

**Methods:**

Qualitative data collection methods were triangulated in the exploratory study to inform the study objectives. In-depth interviews (IDIs) were conducted with 30 men and 20 women based on the inclusion criteria that they have children aged between 6 and 23 months. An additional 10 key informant interviews with stakeholders in nutrition within the study setting were also carried out. Data from the qualitative interviews were captured in audio files with informed consent and permission to record from the study participants. The interviews were transcribed and translated into English transcripts for coding and analysis. Themes were derived from the five levels of the socio-ecological model of human behavior, namely, (i) individual factors; (ii) interpersonal factors; (iii) community factors; (iv) institutional factors guided the analysis.

**Results:**

Findings from the study revealed that personal beliefs and values, the nature of work, mistrust and stigma and discrimination, and the association clinic visits with HIV testing, were some of the factors that influenced male involvement in maternal and child nutrition.

**Conclusion:**

It is important to recognize the potential value of research on the role of men in maternal and child nutrition and to identify ways to overcome the barriers to their involvement. By better understanding the factors that influence male involvement in maternal and child nutrition and the impact of this involvement on maternal and child nutrition, it may be possible to develop more effective interventions to promote the nutritional well-being of mothers and children.

## Introduction

Optimal maternal nutrition and complementary feeding are critical for the health and well-being of mothers and young children. Adequate nutrition during pregnancy is essential for the proper growth and development of the fetus and can help to reduce the risk of complications during pregnancy and childbirth [1–3]. Similarly, optimal complementary feeding during the first two years of life is essential for the proper physical and cognitive development of young children and can help prevent malnutrition and micronutrient deficiency [1]. Investing in maternal and child nutrition during this critical period can have long-term benefits for the health and well-being of both mothers and children. Adequate nutrition during pregnancy and early childhood can help to prevent stunted growth and promote more optimal growth and development, which can have positive impacts on children’s health and educational outcomes. It can also help to reduce the risk of maternal and child morbidity and mortality, which can have a significant impact on the overall health and well-being of families and communities [1, 3].

The 2014 Kenya Demographic Health Survey (DHS) found that 26% of Kenyan children had stunted growth and 8% were severely stunted, while 1% of children were severely wasted and 4% were wasted. These findings indicate that more needs to be done to support optimal feeding practices for young children in Kenya. In addition to poor feeding practices, maternal and child anemia is also prevalent in Kenya, with 36% of children under 5 and 42% of pregnant women suffering from anemia [4]. These nutrition-related problems can have serious and long-lasting consequences for the health and well-being of mothers and children and may be influenced by a range of underlying factors. Haddad's gendered conceptual framework of the determinants of nutrition outcomes provides a useful framework for understanding the complex and interconnected factors that can contribute to poor nutrition outcomes. This framework highlights how discriminatory actions against women, such as unequal access to resources and decision-making power, can have negative impacts on children's health and nutritional status at various levels [5]. For example, unequal access to household resources based on gender norms of resource distribution can make it difficult for mothers to provide their children with adequate nutrition, as they may have limited control over the resources needed to purchase or produce nutritious foods. This can be especially challenging in contexts where women have limited access to education, employment, or other sources of income, and may depend on their partners or other male family members for financial support [5–7].

In Kenya, many large-scale nutrition programs focus on supplementation, fortification, and nutrition education [8–10] and often target women as the primary beneficiaries. These programs may use strategies such as empowerment, nutrition education, and autonomy to improve nutrition outcomes [11, 12]. However, programs that exclusively focus on women may not always take into account the power dynamics within households and how these dynamics may limit women's ability to adopt healthy behaviors and access nutritious foods. This can be challenging in patriarchal settings where women may have limited decision-making power and control over financial resources [13]. Women’s low status in patriarchal settings often places considerable limitations on household decision-making and control over financial decisions and this influences factors such as transport to access health facilities [14] and even access to nutritious foods. To address these challenges and optimize nutrition outcomes for mothers and children, it may be necessary for maternal and child nutrition programs to focus on both women and their partners and to consider how gender dynamics and social norms may influence behavior and access to resources. Working with both men and women can help to challenge harmful patriarchal norms and to promote more equitable and supportive relationships within households, leading to a lasting impact on maternal and child health outcomes in Kenya. This may involve providing education and resources to both men and women and promoting a more equitable distribution of household responsibilities and resources.

### Socio-ecological framework

Several frameworks or models have been used to study nutrition including the socio-ecological model (SEM) [15–17]; however, the model has not been used to understand male involvement in maternal and child nutrition. Our study used the SEM as proposed by Urie Bronfenbrenner in the 1970s [18], to explore factors influencing male involvement in maternal and child nutrition.

The socio-ecological model (SEM) is a framework that can be used to understand the complex and interconnected factors that influence health behaviors and outcomes. It looks at multiple levels of influence. At the individual level, it focuses on individual characteristics such as knowledge, attitudes, and beliefs, as well as personal behaviors and decision-making processes. At the interpersonal level, factors such as relationships with others, such as family members, friends, and peers, as well as social networks and social support are included. The community-level factors include the cultural, social, and physical environments in which individuals live, including expectations and norms within the community. The last level is the institutional level which includes larger societal and cultural influences such as laws, policies, and organizational systems that shape personal behavior. The SEM recognizes that these different levels of influence interact and can have a cumulative impact on health behaviors and outcomes. By considering these multiple levels of influence, the SEM can provide a more comprehensive understanding of the factors that impact health behaviors and outcomes and can inform the development of interventions and strategies to promote health and well-being [18].

Hence, our study used the SEM to explore the factors that influence male involvement in maternal and child nutrition in low-income informal settlements in Nairobi Kenya and how these factors can be addressed to promote more optimal outcomes.

## Methods

This study was part of a larger project (Drivers of Food Choice) and was carried out conducted in peri-urban informal settlements in Nairobi’s Dagoretti North and South sub-county. The area is characterized by low-income residential settlements with a lower population density than other parts of Nairobi [19]. The area also has a higher density of livestock, resulting in a multiplicity of animal source foods (ASF) value chains [19], which were important factors in choosing the study location. The majority of those residing in these informal settlements belong to various ethnic groups, the majority of which follow a patriarchal system in which male household members are viewed as the family breadwinners.

### Recruitment and training of field staff

Two field interviewers, both university graduates and with prior experience in collecting qualitative data, were recruited and trained for four days. The training content included the objectives of the study, qualitative data collection methods, interview guides, and ethics in research.

### Recruitment of study participants and data collection

The study utilized qualitative in-depth interviews (IDIs) and key informant interviews (KIIs) to inform the exploratory study design. A total of 50 participants; 30 men and 20 women were recruited through purposive sampling taking into consideration ethnicity, the village of residence, and age. To be eligible for the study, participants had to be men and women of reproductive age recruited from different households with children between 6 and 23 months of age and willing to voluntarily participate in the study.

In-depth interviews (IDIs) were conducted to understand the individual, interpersonal, community, and institutional level factors influencing male involvement in maternal and child nutrition in resource-limited settings. Telephone calls were used to conduct the in-depth interviews. The contact information was obtained via participant lists created using records or respondents generated for the wider study in 2019 and 2020. Potential interview subjects were contacted before the interviews to arrange a suitable time and day and verbal consent was obtained.

Community health workers helped with the recruitment of the key informants consisting of community health volunteers [6], clinicians [2], and nutritionists [2]. These were individuals who had lived or worked and interacted with the study communities and had a good understanding of male involvement in maternal and child nutrition. The aim was to gain an overview of the attitudes and practices of men concerning maternal and child nutrition.

The content of the IDIs and KIIs was framed with open-ended questions guided by the SEM and addressed the following issues: individual, interpersonal, community, and institutional factors. Given that participants came from different backgrounds, interviews were conducted in Swahili, the commonly used language in the study area.

### Ethical considerations

The ethical approval for the study was obtained from the National Commission for Science, Technology, and Innovation (NACOSTI/P/20/7426). Further, all the study participants were briefed about the purpose of the study and the right to refuse to answer any question or withdraw from the interviews at any time. They were also informed that there were no “right” or “wrong” answers and were requested to express their opinions and thoughts freely. They were informed that their names will be kept strictly confidential and that their names will not appear in any publication or report resulting from this study. Before any data collection, detailed verbal informed consent including permission to record the interviews was sought for all the participants.

### Data analysis

Interviews were audiotaped and transcribed verbatim. Two graduate students who had been trained performed simultaneous transcription and translation. The transcribed word files were transferred to QSR NVIVO 11 software for coding. Coding and interpretation were done by two members of the research team to ensure comprehensiveness and reliability in the application of the coding process. Final checks for consistency of the application of the codes were undertaken by a third member of the research team. Following the completion of coding, related codes were grouped into categories, and themes derived from the five levels of the socio-ecological model of human behavior, namely, individual factors; interpersonal factors; community factors; and institutional factors.

## Results

The results are presented based on the different themes entailed in the socio-ecological model.

### Individual-level factors influencing male involvement

### Personal beliefs and values

Male involvement in maternal and child nutrition may be influenced by their own beliefs and values about the importance of family and the role they should play in supporting the health and well-being of their household members. It was clear that men did not perceive nutritional issues as their responsibility. These men were distant from routine nutrition activities and only felt responsible for income-generating activities. In this role, they perceived that they were giving full priority to their family because they were fulfilling their responsibility as needed. These men cited the provision of finances for purchasing food as a key contribution they made to the family as exemplified in the following excerpts:

I provide her with what she requires since she spends most of the time with the child …. I am 100% involved in their welfare. **(IDI Men 24)**

When my wife was pregnant, I ensured that I provided all the things she required such as food and transport money for clinic visits **(IDI Men 24)**

The view of men as providers of their families was generally confirmed by women respondents as shown below:

My husband leaves the responsibility to me so that I take care of the baby, he provides the money and leaves the other responsibility to me.** (IDI women 26)**

Most men mentioned assisting with logistics, finances, and accompaniment to the clinics when it came to maternal and child healthcare. Most men viewed health care, particularly pregnancy and childbirth, as a female domain. Only when it was completely necessary would a man accompany their wife to the clinic, but regular visits were considered a waste of time. One man in an IDI indicated, *“Because I have twins, I must help her by accompanying her to the clinic occasionally.”* It is also notable that the few men who accompanied their wives to clinic appointments did not necessarily go into the healthcare facility or see the doctors with their wives. In an IDI, a woman indicated, *“When I went with my husband, he would escort me to the gate.” This was corroborated by a CHV. “When they go to the clinic[*men*] they stay out and wait for the woman at the gate. Most men miss the information in this way.”*

### Knowledge of maternal and child nutrition

Most women noted that their husbands had limited knowledge of what constitutes nutritious food for pregnant women or young children and that they primarily focused on bringing home food regardless of its nutritional value.

He doesn’t know anything about it, he just cares that people eat and they are full. He.

knows something small about a balanced diet but he doesn’t understand it deeply. **(IDI Women 34)**

This highlights the importance of providing education and resources to both men and women on the importance of maternal and child nutrition and the specific nutrients and foods that are necessary for optimal health. By increasing knowledge and understanding of maternal and child nutrition, it may be possible to encourage more supportive and nurturing behaviors within households and to promote more optimal health outcomes for mothers and children. This may involve providing education and resources to men specifically, as well as working to challenge harmful patriarchal norms and promote more equitable and supportive relationships within households.

### Nature of work

From the study, time and the nature of work were significant factors that did limit male involvement in maternal and child nutrition. Many men in the study mentioned that long working hours or shift work left them with little time to be actively involved in maternal and child nutrition. Work demands also left men unable to devote attention to maternal and child nutrition. Participants explained that prioritizing such responsibilities often came at the cost of direct involvement in household nutrition. This was captured in the following quotes:

Most[of us men] are at work, in places where if you ask for permission from work constantly then you can be fired. I think there is also embarrassment when some men want to get involved in doing such things, they just want to be able to provide food. **(IDI Men 31)**

My job requires me to work long hours and often on weekends, so I don't have much time to be involved in my family's health and nutrition. I wish I could do more, but it's just not possible with the demands of my work. (**IDI Men 30)**

However, there seemed to be a bit of disagreement on this as exemplified by this excerpt:

It’s not even an issue of time, he could be at home after working night shifts, but I don’t know why he does not want to go to the clinics** (IDI Women 21)**

However, not all men were hindered by the nature of their work, but rather cited other reasons for their limited involvement. These reasons included a perception that it was a "waste of time":

I already have enough on my plate; I don't have time to waste it on something that won't change anything **(IDI Men 33).**

It is just not a priority for me. There are more pressing matters that I need to focus on **(IDI Men 30).**

The men also indicated the fear of being asked difficult questions if they accompanied their wives to appointments.

I am afraid that the doctors will ask me questions that I don't know the answers to, so I don't want to attend appointments with my wife **(IDI Men 33).**

I don't know a lot about maternal and child nutrition, and I am worried that if I ask questions, I'll come across as ignorant** (IDI Men 24).**

### Mistrust and suspicion in marriage

It was discovered that a barrier preventing men from attending their spouse's clinic appointments was sentiments of mistrust and suspicion, which were increased by the belief that their partner was hiding something, such as infidelity. Some men found it uncomfortable to be seen by others and this made the men less inclined as a result to bring their partners along to clinic sessions. “You may find that the man has another young woman outside and so doesn’t want to be seen that he has a wife.” **(IDI Men 29).** This was corroborated by women respondents. “I also don’t trust him, if he can’t walk with me because there could be something he is hiding, like that his wife is pregnant and maybe he doesn’t want to be seen by one of his women” **(IDI Women 20).**

### Interpersonal level factors influencing male involvement

From the study, two main interpersonal factors influenced male involvement in maternal and child nutrition. These were: relationship dynamics and communication patterns within the household and social support. Concerning relationship dynamics and communication patterns, men expressed that a lack of understanding of nutrition matters made it difficult for them to feel motivated or supported in their involvement. Conflict within the household also made it difficult for men to feel comfortable in involving themselves in maternal and child nutrition. Here, men expressed difficulties in getting “too involved” in matters of nutrition due to a desire to avoid confrontation. “Too involved” meant doing additional roles to their work as economic providers such as offering advice or suggestions on what to prepare, preparation of meals, and sometimes purchasing ingredients required for meal preparation. The men noted that too much involved may cause problems at home as per these excerpts:

He may rub shoulders with his wife because his wife will think that he is showing her that she does not know how to do her job. Also, the man might concentrate too much and neglect his responsibility to provide**. (IDI Men 33)**

Yes, people can talk [about a man taking a child to the clinic] but by taking the baby to the clinic himself the man can find out that the woman was asking for more money because she does not have a source of income so she can use that as a reason to get more cash which can lead to the breakup of the family. **(IDI Men 25)**

Women and community health workers also felt that direct engagement by men would be a cause of conflict in the house giving different reasons as exemplified in these excerpts:

The wives may get angry because they want to do the budget since some may want to set aside some money for themselves**. (IDI Women 46)**

There might be conflicts because the woman thinks she knows more about what the child needs. So, you find that in a household there are some disagreements when a man tries to suggest something. It might look to the woman that they are competing. **(KII CHV)**

In our community, you will be told that you are competing with your wife over such embarrassing issues **(KII Clinician)**

### Community-level factors influencing male involvement

#### Stigma and discrimination

Stigma and discrimination can have a significant impact on male involvement in maternal and child nutrition. Men who take an active role in this area may face negative stereotypes and be viewed as challenging traditional gender roles. This can lead to social isolation which can discourage men from continuing to be involved in maternal and child nutrition. Men who were seen to be engaged in “women’s business” were stereotyped and gossiped about by fellow men and women. This resulted in negative views such as men being controlled and manipulated by their wives. Some were also seen to be mean as they wanted to control the spending of money meant for the household and others were also seen as being idle. On the other hand, women who allowed their husbands to engage in “their work” were seen as lazy:

People will say that the man does not want to give the woman money, he is mean. The man buys the food himself; he sees as if he gives the woman money, some will be left, and the woman will not reveal this but keep it**. (IDI Men 43)**

People will say that the man is controlled, and when the man hears that, he thinks that if he goes there [to the health facility], he will be not respected, and people will think that he is being controlled**. (KII Nutritionist)**

Men fear how their image will be perceived in the community and especially by their peers and friends. They also have pride because they think women are controlling them. That makes them feel as if their manhood is violated. **(KII CHV)**

However, attitudes especially in the younger generation were beginning to change as men try to play a more active role in the different aspect’s household nutrition:

Men in their twenties are the ones who cooperate and are willing to adopt new practices., The older men don’t usually cooperate, especially if the man works at night, they don’t want to be involved in what they consider the mothers’ responsibility **(KII CHV)**

There are challenges from his peers and relatives to stop doing a woman’s job, but he doesn’t listen to them, I like him because he loves me, and we are together by God’s grace and we take care of our family. **(IDI Women 24)**

### Institutional-level factors influencing male involvement

#### Association with HIV testing

From the study, accompaniment to the clinic was highly associated with HIV testing. Among the members of the community, it is common knowledge that if you go to the clinics, HIV testing is mandatory. This idea stems from earlier Prevention of Mother to Child Transmission of HIV (PMTCT) campaigns that urged men to be tested for HIV at local health facilities.

Men are afraid of knowing their HIV status because when the spouse is pregnant you must have your HIV status checked, so they don’t want to keep on going to the clinic **(IDI Men 46)**

He fears how people will talk because when I went with him to get an HIV test, we went together. **(IDI Women 35)**

#### Reception at the health facility

From the study, it was clear that it was mainly women that took children to the health facilities during clinic appointments. Men who did accompany their wives to the clinic reported receiving good treatment from the staff. By providing a welcoming environment and ensuring that men feel valued and respected, healthcare providers encouraged men to be more involved.

There was a time when I went with my wife to the clinic and I noticed other men in the queue also. . I remember that we were served faster as couples than the others who came alone **(IDI Men 31)**

The reception was very good the first time. I was informed that most men don’t want to accompany their spouses. So, I was encouraged **(IDI Men 24)**

Men noted that their involvement in maternal and child nutrition specifically through clinic attendance was essential as it would enable them to get firsthand information that was more valid.

At times it is difficult to trust your spouse when she tells you about the items, she has been advised by the doctor to buy. But when you get firsthand information from the doctor you have no other choice but to accept. **(IDI Men 31)**

It will be easier for the husband to provide without any problem[if he gets information firsthand from the clinic]. If it comes from the wife, he may refuse because he sees it as a luxury. **(IDI Men 30)**

Women further reported that if men received information directly from the clinics, they would take issues of nutrition as being important:

From what we are told at the clinic, I may want to follow that and buy something but the money to do that is not there. If he went to the clinic and heard what we were being told to do, then he would fully provide. **(IDI Women 45)**

It is good when he is involved because he gets to hear from nutrition specialists and that will make him buy the foods that they recommend. **(IDI Women 23)**

It is good for him to be involved since he gets to know the price of those types of food. So that he doesn’t think his wife may be lying to him or that the money is used for other purposes **(IDI Women 24)**

## Discussion

Men are in a unique position to improve maternal and child nutrition in their households and the broader community because of their power and authority. Men often make key decisions about how financial resources are to be used in the household which in turn influences a household’s dietary intake [20]. As illustrated in this study, men saw the provision of material and financial support as one of the most important ways that they participated in ensuring proper maternal and child nutrition whether by providing money to buy food at the household as well as paying for clinic appointments and facilitating clinic visits. Respondents agreed that men partners are the primary decision-makers on household finances even on issues of purchase of food. Women on the other hand made decisions about what foods to buy and how to prepare the food with funds given by their husbands. Men were gatekeepers of finances and controlled its use whether for purchasing food or visiting clinics. Some women felt unable to make nutritional changes without the support of their male partners as men held some power over household nutrition because of the financial assistance they provided. Similar findings were reported in several studies where mothers reported control over cooking decisions but did not feel empowered to make nutritional changes that included additional costs. In this study, the women’s incapability prevented her from demanding more than the usual allowance that she is given for acquiring cooking ingredients. In a study in Burkina, Faso women had limited financial autonomy and insufficient support to carry out practices related to nutrition effectively. Women described themselves as not being able to buy the food they required and therefore had to depend on men allocating them resources that were often inadequate to cover household needs [21, 22].

Our findings suggest the existence of strong social and gender norms regarding the duty of men to care for their household members in the community. While this is seen as a very important part of men's roles, some aspects are seen as the domain of women, particularly those relating to maternal and child nutrition. Most men indicated that they were not directly involved in maternal and child nutrition due to competing job priorities. Although men highlighted many reasons for direct engagement in household nutrition, the majority highlighted that they were mostly constrained by their work obligations. Men emphasized that although they never directly participated in accompanying their wives to clinic appointments, they often provided money for the appointments and transport to the clinic. It was clear that by missing out on clinic appointments, men often missed opportunities to access appropriate messages about nutrition from trained health workers. Similarly, a study in Ethiopia related to maternal health issues revealed that inadequate knowledge of men partners about maternal and child health issues was a huge barrier to attending skilled antenatal care, where health talks about appropriate child-feeding practices are usually given [23]. Through these findings and those of our studies men accompanying their wives for clinic appointments is an important part of their involvement in their household nutrition. Although men felt that employment was a structural determinant, some women disagreed with this as some of the men worked night shifts and were therefore available during the day for clinic visits. This finding is similar to that of Anguzu et al. [24] that revealed that men’s occupation did not influence clinic attendance as 64% of 286 manual workers frequently attended antenatal visits with their spouses.

From the study, men who were seen to embrace active involvement in maternal and child nutrition are seen to be under control by their wives. This is due to the traditional gender roles that place men as breadwinners and women’s caretakers of the family. This was attributed to rigid roles in different cultures associated with male engagement in specific domains within the household. Traditional gender roles and social stigma had a negative effect on men’s involvement and were among the major obstacles in this regard. For example, men often did not want to engage themselves in activities such as accompanying their wives to the clinic due to community perceptions. A study by Vermeulen et al. found that although men knew that prenatal cares were important for pregnant women, they did not take part in direct involvement in this regard and attributed their inaction to external factors [25]. In another study by Asefa et al., men referred to their feeling embarrassed in accompanying their wives as the major obstacle to their participation [23]. From our findings, mistrust and suspicion in marriage also had a negative impact on a men's willingness to attend clinics. If a man feels that his wife does not trust him or suspects him of infidelity, he may feel uncomfortable or resentful about going to a clinic, even if it is important for the health of the mother and child. Additionally, if a man feels that his wife is overly controlling or critical of his actions, he may be less likely to see that accompanying his partner to the clinic, it is not worth the potential conflict.

Further, from the study, male attendance in clinics was associated with HIV testing which kept men away from health facilities. This is related to the fact that most male involvement programs have focused narrowly on men’s involvement in PMTCT [26] and family planning [27]. This narrow focus can lead to the stigmatization of men who accompany their wives for clinical appointments, as they may be perceived as only being present for these specific purposes, rather than for general maternal and child health care. This can discourage men from attending clinics and seeking care for themselves and their families, which can have negative impacts on maternal and child health.

### Limitations

There are two limitations to this study. First, the cross-sectional exploratory design of this study means that the causal relationship between male involvement and optimal maternal and child nutrition outcomes could not be established. Future research is required to comprehend and validate the associations found in this study. Second, the sample size used in the study was small. Future studies using larger sample sizes are needed to enhance the generalizability of the findings. The study findings do, however, offer valuable qualitative data on male involvement in maternal and child nutrition in low-income settings from which future studies can use as a starting point.

## Conclusion

Male involvement in maternal and child nutrition is important because men can play a crucial role in supporting the health and nutrition of their families. Men’s attitudes and behaviors toward nutrition can have a significant impact on the dietary choices of their families. In patriarchal settings, men are the primary decision-makers when it comes to the household budget and food purchasing, so their involvement can have a significant impact on the nutritional status of the household.

In addition to these practical considerations, male involvement in maternal and child nutrition can also help to promote gender equality within the household. When men are actively involved in the care and feeding of their children, it can help to reduce the burden on women and improve the overall well-being of the family. By challenging traditional gender roles and promoting more inclusive and equitable roles for men and women, programs can help to create a more supportive and healthier environment for families.

### Recommendations

Men are essential for ensuring optimal nutrition; hence, it is crucial to include them as vital participants in maternal and child nutrition programs. Some of the measures would entail identifying local male role models and peer educators. By identifying men who are actively involved in maternal and child nutrition and who are respected in the community, programs can leverage their influence to promote greater male participation.

Another key consideration would be engaging men in nutrition education programs. By providing men with accurate and relevant information about the importance of their involvement in maternal and child nutrition, programs can help to dispel myths and encourage more men to participate.

Programs could also work to understand and respect cultural norms, while also promoting more inclusive and equitable gender roles. This may involve working with local leaders or community organizations to promote change.

Another critical consideration would be engaging men in decision-making. Programs can involve men in the planning and implementation of maternal and child nutrition initiatives, which can help to build buy-in and encourage greater participation.

Overall, programs need to take a holistic and inclusive approach to involve men in maternal and child nutrition and to work to address any barriers or challenges that may prevent men from participating.

## Data Availability

The datasets used for the article and the study are not publicly available because personal confidentiality may be compromised, but data and materials can be obtained from the corresponding author upon reasonable request.

## Abbreviations

ASF: Animal source foods
CHV: Community health volunteers
DAGAS: Department of Anthropology Gender and African Studies
IDI: In-depth interviews
KII: Key informant interviews
PMTCT: Prevention of Mother to Child Transmission of HIV
NACOSTI: National Commission for Science, Technology, and Innovation
SEM: Socio-ecological model
UoN: University of Nairobi
